# Intracellular Signaling Mechanisms Pharmacological Action of Jasminum amplexicaule Buch.-Ham. (Oleaceae) on Gastrointestinal Secretion 

**Published:** 2014

**Authors:** Zhenhua Gao, Junqiang Yin, Xiaolin Xie, Hanwu Long, Xiang Qi, Changhu Lin, Liangcai Wu

**Affiliations:** a *The First Affiliated Hospital of Sun Yat-Sen University, Guangzhou 510080, China.*; b *The Insititute of Biology, Guizhou Academy of Sciences, Guiyang 550009, China. *

**Keywords:** *Jasminum amplexicaule*, T_84_ cell, Secretion, Anti-diarrhoea

## Abstract

Jasminum amplexicaule Buch-Ham. (Oleaceae) has been commonly used in the traditional medicine in dysentery, diarrhoea and bellyache in China. In the present work, the methanol extract of *Jasminum amplexicaule *(JME) was examined for pharmacology on human colonic epithelial cell line T_84_ by the short-circuit current technique. The results showed that pretreatment of T_84_ cells with JME produced a concentration-dependent (0-1000 μg/mL. EC_50_ = 0.055 mg/ mL) inhibition effect on adrenalin (Adr.)–induced Cl- secretion. The maximal response was observed at 200 μg/mL. It has been demonstrated that JME has a direct effect on the enterocyte. Our results also demonstrated that the JME exerted inhibitory effect on gastrointestinal Cl^-^secretion that effected by acting on basolateral β-adrenoreceptors. These results suggested that the Chinese traditional medicine of JME can be used for the treatment of acute diarrhea and bellyache.

## Introduction

*Jasminum amplexicaule *Buch.-Ham. (Oleaceae) is a kind of traditional medicine mentioned above, which is a genus of about 600 species of shrubs and small trees in the family Oleaceae. It is a multipurpose plant with great potential for ethno medicinal application in some parts of China and India. The powder of its twigs and leaves has been used as a hydragogue and a febrifuge and also has been used as a kind of traditional medicine for the treatment of dysentery, diarrhoea and bellyache in China (1). Its leaves are used to take care of the quadriplegia-gall and also mixed with other ingredients to cure dysentery and bellyache (1). A phytochemical study reported that four secoiridoids glycosides were isolated from the dried twigs and leaves parts of *J. amplexicaule *(2, 3). A report studied its anti-diarrhoea and analgesic activities (1). The anti-diarrhoea activity can be ascribed to inhibition of intestinal motility and/or the anti-secretory properties (4) on gastrointestinal (syn GI) tract. 

It is well known that, as the first defense line, the GI epithelium of the host plays an important role in protecting enteric epithelia from invasion of most pathogens. Intestinal epithelial barrier function regulates epithelial ions and nutrient transport as well as host defense mechanisms. Epithelial membrane pumps, ion channels and tight junctions control epithelial transcellular and paracellular fluxes (5, 6). Epithelial Cl-channels play an important role in regulation and maintenance of normal GI physiological functions. Abnormal regulation of Cl^-^ channels may result in diarrhea (7, 8) or constipation (9). 

Because Cl^-^ provides an essential driving force for lubrication of intestinal contents during regular bowel movements or artificial irritants in host defense responses (10), Cl^-^ channels are very likely to be the targets for pharmacological intervention. Our present study focuses on examining whether ME exerted any effects on inhibition Cl^-^ secretion and investigating which mechanism was involved in.

## Experimental

*Plant material and extraction*

The twigs and leaves of *Jasminum amplexicaule *were collected from Botanical Garden of Department of Medicinal Plants, Southern Medical University (the first military medical university) in Guangzhou in September 2008 Guangdong, and identified by Prof. Qianhai Chen (Guizhou Academy of Sciences, P. R. China). Voucher specimens and samples were stored at the Institute of Biology, Guizhou Academy of Sciences. The methanol extract (JME) was filtered and evaporated to dryness using vacuum rotary evaporator (yield 14.2%). At the time of using, drugs tested were freshly prepared in the distilled water.

*Cell culture*

Human colonic T_84_ cells (American Type Culture Collection) were maintained in Dulbecco’s modified Eagle’s medium (DMEM)/F-12 supplemented with 10% (v/v) fetal bovine serum, 1% (v/v) nonessential amino acids, 100 IU/ml penicillin, and 100 μg/mL streptomycin. For simultaneous measurements of I_SC_, the cells (1.5×10^6^/mL) were seeded onto a filter (Millipore, U.S.A) with a silicone rubber ring on top of it to confine the cells (0.45cm^2^). Cells reached confluency after 8 to 10 days, with a resistance greater than 300 Ω/cm^2^.

*Short-circuit current measurement*

The measurement of I_SC_ for human colonic T_84_ has been described (11). The monolayers grown on permeable supports were clamped vertically between two halves of the Ussing chamber and both sides bathed in normal Krebs-Henseleit solution (K-H solution, pH 7.4) while the I_SC_ was measured. The K-H solution maintained at 37 °C by a water jacket enclosing the reservoir. The solution was bubbled with 95% O_2_ and 5% CO_2_ so that the pH of solution was maintained at 7.4. Drugs could be added directly to the apical or basolateral side of the epithelium. Electrodes for measuring transepthelial potential difference (PD) and passing current were connected to the chamber. The transepithlial PD were then clamped at 0 mV, and the short circuit current was recorded with VCC MC6 voltage–current clamp amplifier (Physiologic instrument, San Diego, CA) and displayed using a signal collection and analysis system (BL–420E, Chengdu Technology & Market Co. *Ltd, *China). The change in I_SC_ was defined as the maximal rise in Isc following agonist stimulation and it was normalized to current change per unit area of the epithelial monolayer (μA/cm^2^). Transepithelial resistance (Rt) was calculated by measuring the current response to a 1mV pulse. The Isc value is expressed as positive when the current flow from mucosal to serosal.

*Solutions*

The bicarbonate-buffered Krebs-Henseleit solution contained (mM) NaCl, 117; NaHCO_3_, 25; KCl, 4.7; MgSO_4_, 1.2; KH_2_PO_4_, 1.2; CaCl_2_, 2.5; and D-glucose, 11, pH 7.4, when bubbled with 5% CO_2_, 95% O_2_. The bath temperature was maintained at 37 °C using a heated water jacket. Adrenalin, nystatin, Carbachol and propranolol were obtained from Sigma. Stock solutions of all the chemicals were dissolved in DMSO. Final DMSO concertration never exceeded 0.1% (v/v).

*Statistics*

The data were expressed as X ± S.D. Concentration-response relationship was calculated with Hill equation. Statistical significances were analyzed by Student’s t-test. p*-*value < 0.05 was considered significantly.

## Results

*JME-induced anion secretion*

**Figure 1:**
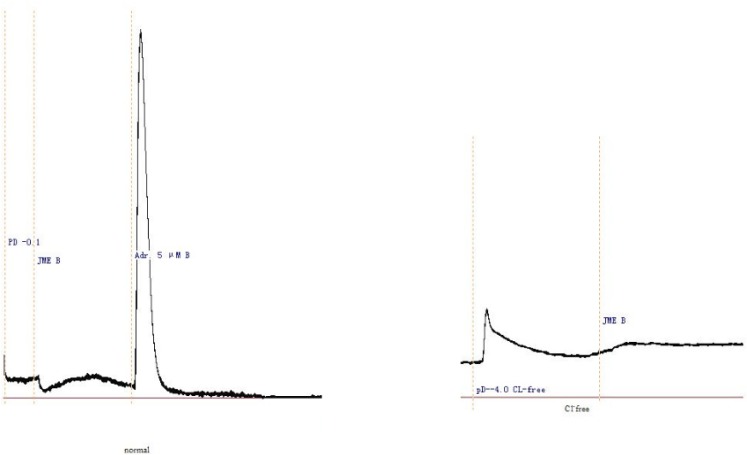
Representative Isc recording with arrows indicating the response to JME (0.05 mg/mL) added in normal and Cl- free K-H solutions

JME elicited a significant secretory response when added to the basolateral side of T_84_ cells at a concentration-dependent manner, as shown in Figure 1. In order to study the ion species involved in mediating JME induced *Isc*, a series of ion substitution experiments were conducted, Cl^-^ was removed from the bathing solution. The JME-induced Isc response was completely abolished by removing Cl^-^ from bath solution (Figure 1), indicating that the JME-induced Isc was almost induced by Cl^-^. 

*Short-circuit current measurement effect of JME on secretagogue-induced Cl* ^-^ *secretion*

**Figure 2:**
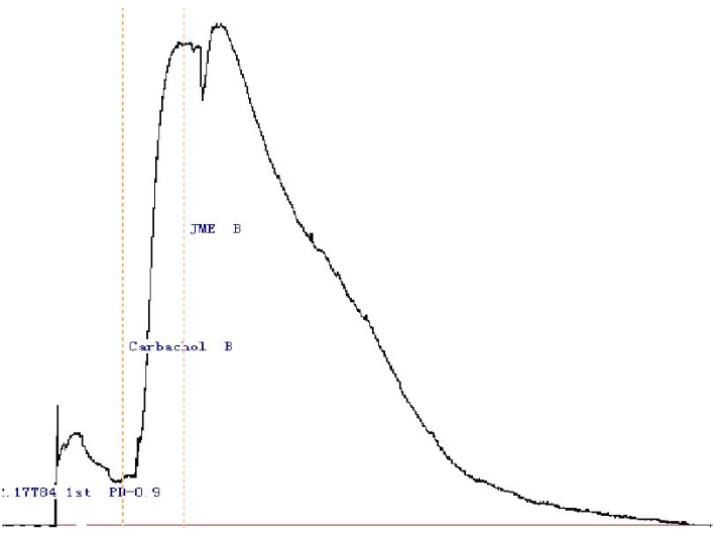
Representative Isc recordings of the T_84_ cell line in normal K-H solution. Stimulation with Carbachol (100 μM) on basolateral side resulted in an increase in Isc, which was not inhibited by either side application of JME (0.2 mg/mL) (n=3) .The time of the drug administration is indicated with arrow

JME concentration of 2.0 mg/mL was selected to study its effect on secretagogue induced Cl^-^secretion, since at this concentration application of JME can not induce ion transport on either side of the monolayers. In Figure 2, stimulation with Carbachol (100 μM) on basolateral side resulted in an Isc increase; application of JME (0.2 mg/ mL) cannot further induced the Isc increase on either side of the monolayer (Figure 2). 

**Figure 3:**
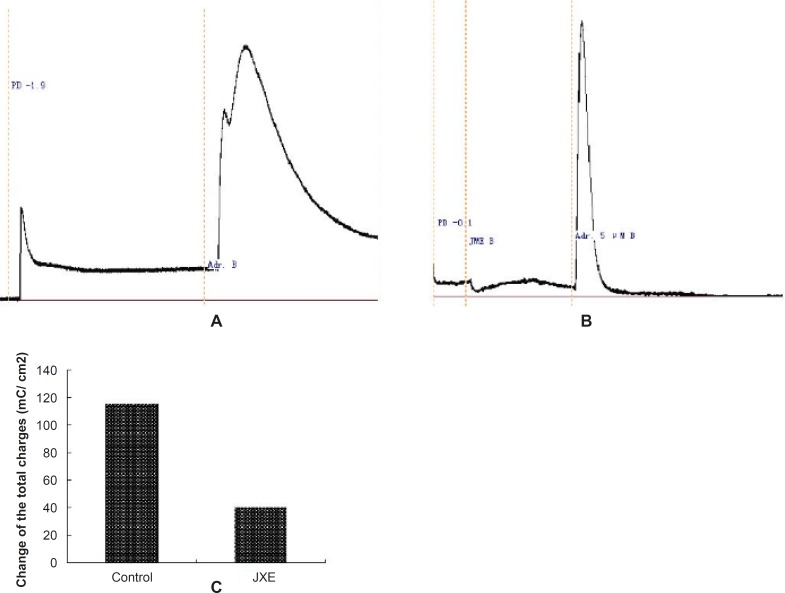
Representative Isc recordings of the T_84 _cell line in normal K-H solution. (n=3).

In Figure 3, the basolateral added adrenalin (5 μM, Adr.) produced a significant Isc increase, which lasted for at least 30 min. In order to study the ion species involved in mediating the Adr.- induced Isc, Cl^-^ was removed from the bathing solution. The response was completely abolished by Cl^-^ removal, indicating a Cl^-^ dependence of the Adr.-induced current. The JME (0.2 mg/mL) had also no significant effect on Adr.-induced Cl^-^ secretion on either side of the monolayer. Pretreatment of T_84_ cells for 20 min with JME (0.2 mg/mL, either side) had no significant effect on carbachol-induced Cl- secretion. Pretreatment of T_84_ cells for 20 min with JME (0.2 mg/mL, apical) had also no significant effect on Adr.- induced Cl^-^ secretion. In contrast, when T_84_ cells were pretreated with JME (0.2 mg/mL, basolateral) for 20 min, the second area secretory response to Adr. was inhibited significantly. 

A: Representative Isc recordings in response to adrenalin of the T_84_ cell line in normal K-H solution.

B: T_84_ cells have preatreated with JME (0.2 mg/mL, basolateral) for 20 min before adding adrenalin.

C: Comparison between A and B in total charges.

**Figure 4:**
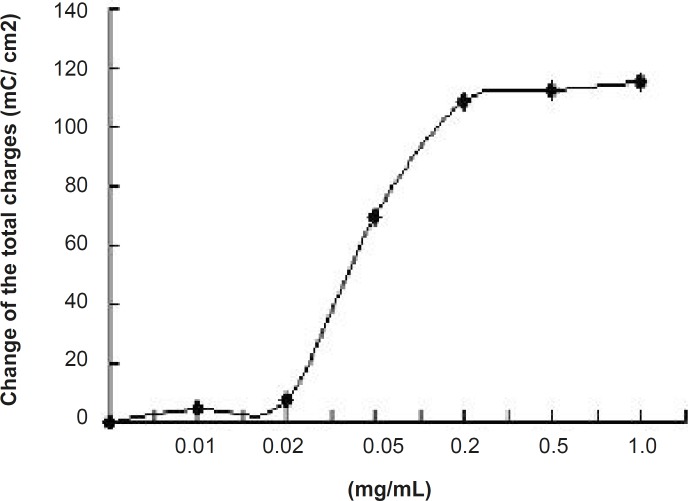
Concentration- response curve for JME-inhibited Isc was in T_84_ cell line. Values are mean ± S.D. of maximal Isc decrease (n=3).

**Figure 5:**
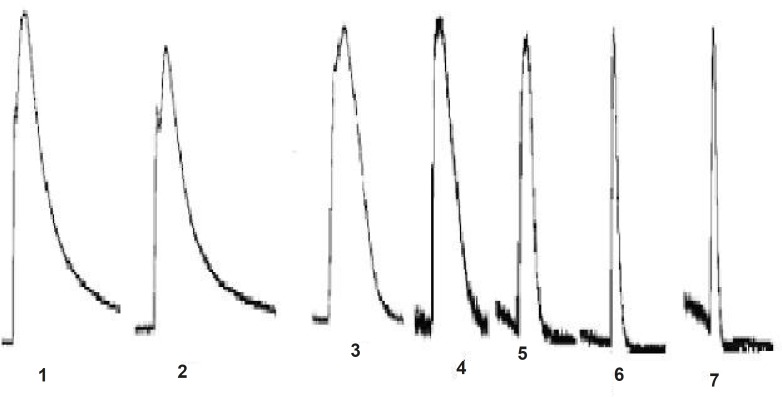
Representative Isc recordings in response to JME of the T_84_ cell line is in normal K-H solution (n=3).

The effect of JME extract was concentration-dependent (Figure 4) with EC_50_ of about 0.055 mg/mL. Different concentrations of JME are added to basolateral sides (Figure 5). 

## Discussion

The present study is the first to investigate the pharmacological action of JME on intracellular signaling mechanisms. JME exerted a significant secretory effect at the higher concentration assayed. According to JME’s traditional using, the mechanism of JME on secretagogue-induced Cl- secretion was selected for the subsequent experiments. 

It has been reported that adrenalin induced Cl^-^ secretion in some epithelial cells through action of basolateral adrenoreceptors, such as epididymal epithelium and colonic cells (12). In normal Krebs solution, Adr. elicited a biphasic Isc response consisting of an initial spike followed by a delayed, but more sustained response. Pretreatment of T_84_ cells for 20 min with JME (0.2 mg/mL, basolateral) can abolish the second response. Using the nonselective β-adrenoreceptors antagonist propranolol can aslo abolish the components. It is suggested that JME exert a direct inhibitory effect on basolateral β-adrenoreceptors so that the opening of the apical anion channels would be somehow compromised. The inhibitory effect of JME extract was concentration-dependent. 

It is interesting to find that, JME was ineffective in blocking the Isc responses to carbachol. It has been reported that in T_84_ cells, carbachol can stimulate a Ca^2+^ dependent Cl^-^ secretion through activation of basolateral M-Ach recptor (13). The present results suggested that ME was ineffective in blocking basolateral M-Ach receptor in T84 cells. 

**Figure 6:**
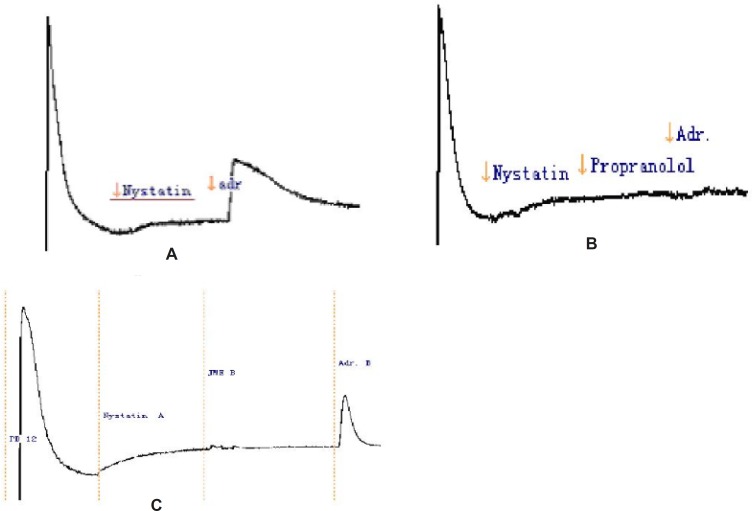
Representative Isc recordings in response to JME of the T_84_ cell line in a low Cl^-^ solution (n=3).

We investigated the putative inhibition of secretion through blockade of basolateral K^+^ channels by using nystatin-permeabilised human colonic mucosae in order to enucleate the effect of I_k_ to JME. Under these conditions of the low Cl- solutions, mucosa to serosa K^+^ gradient, apical permeabilisation with nystatin (180 μg/μL), a relationship between Adr. (5 μM) and K^+^ gradient was observed (Figure 6a). The result showed that Adr cann’t stimulate K^+^ secretion. Under the same condition we added the propranolol (the inhibitor of Adr.,10 μM) on the basolateral of the monolayer, then, the Adr.( 5 μM) was added (Figure 6b). I_sc_ was completely inhibited. So, in the T84 cell line, Adr. induces secretion through activation of basolateral β-adrenoreceptors. Under the same condition when pretreatment of T_84_ cells for 20 min with JME (0.2 mg/mL, basolateral), the second secretory response of Adr.-induced was significantly inhibited (Figure 6c). 

A : I_k_ recorded ,firstly added with nystatin, secondly added with Adr. to the apical side;

B : I_k_ recorded ,firstly added with nystatin, secondly added with propranolol, thirdly added with Adr. to the apical side;

C: I_k_ recorded, firstly added with nystatin, secondly added with JME, thirdly added with Adr. to the apical side.

Taken together, the present results have demonstrated that JME exerted an inhibitory effect on gastrointestinal Cl^-^ secretion that is effected by acting on basolateral β-adrenoreceptors. The ability of JME to inhibit Cl^-^ secretion in the GI tract can contribute to its beneficial effects, such as reducing smooth muscle movement and decreasing the frequency of defecations. 

At the moment, it is uncertain whether the inhibitory effect of JME on Cl^-^ secretion is due to the collective effect of all the constituent herbal components or some active ingredients contained in the JME. Nevertheless, the current study has established a model for the quantitative measurement of JME effect on the GI tract and for further screening of its possible active ingredients. The study of intracellular signaling mechanisms validated the JME pharmacological action. 

The mechanism of action of the JME is still not fully elucidated, but it involves a direct effect on the enterocyte. The effects are not specific for a particular signal transduction mechanism, and may be involved in the anti-secretory effect. The intracellular signaling mechanisms of JME much more validated the JME pharmacological action.
